# Persistence of protozoan parasites and *Escherichia coli* on plant tissue in soil

**DOI:** 10.1128/aem.02467-25

**Published:** 2026-04-30

**Authors:** Kyle J. McCaughan, Michelle D. Danyluk, Kalmia E. Kniel

**Affiliations:** 1 Department of Animal and Food Sciences, University of Delaware 736112, Newark, Delaware, USA; 2 Department of Food Science and Human Nutrition, University of Florida, Lake Alfred, Florida, USA; University of Georgia Center for Food Safety, Griffin, Georgia, USA

**Keywords:** *Cyclospora*, herbs, soil, food safety, protozoa

## Abstract

Tilling under is a method growers employ in an effort to remediate instances of widespread contamination in fields of crops, but its effectiveness with respect to specific pathogens remains poorly understood. This study had two overarching goals: first, to assess the survival dynamics of *Cryptosporidium parvum* and *Eimeria tenella* (used as a surrogate for *Cyclospora cayetanensis*) oocysts, and *Escherichia coli* TVS355 in soil following inoculation onto plant tissue and a simulated “tilling under” process; and second, to evaluate the potential of monitoring mRNA levels over time as oocysts die off as an alternative to traditional infectivity-based methods to determine oocyst viability. Basil, cilantro, and soil without plant tissue were inoculated with *C. parvum* (10^6^ oocysts), *E. tenella* (10^6^ oocysts), and *E. coli* TVS355 (10^8^ CFU) and then incorporated into 120 g of soil; samples were then incubated at 12°C or 32°C for up to 12 months. Monthly, samples were removed and assessed for organism survival, *E. coli* TVS355 through traditional dilution and plating, and oocyst decay through two methods: cell culture followed by qPCR, and mRNA extraction followed by RT-qPCR to measure gene expression. *E. coli* remained detectable in samples for 10 months, and, through infectivity methods, *C. parvum* and *E. tenella* remained infectious for 9 months. However, results from mRNA methods indicated that oocysts remained detectable for at least 12 months, with approximately 1.22 log oocysts/sample detected at that point for both *C. parvum* and *E. tenella*. Temperature impacted *E. coli* TVS355 and *E. tenella* recovery (as determined by infectivity methods) only after 7 months, when slightly more of each organism was recovered from samples incubated at 12°C. Meanwhile, mRNA methods detected significantly (*P* < 0.05) fewer oocysts of both *C. parvum* and *E. tenella* in samples incubated at 32°C as compared to those incubated at 12°C throughout the experiment.

**IMPORTANCE:**

Contaminated crops may be tilled under and buried in the soil, which may lead to the decay of contaminating microbes. This study looked at three microbes that can contaminate produce: *Escherichia coli* (a bacterium), *Cryptosporidium parvum* (a zoonotic protozoan pathogenic parasite), and *Eimeria tenella* (an avian protozoan parasite used as a stand-in for the human parasite *Cyclospora cayetanensis*). Inoculated basil and cilantro were mixed in soil and stored at 12°C and 32°C for 1 year. Both protozoan parasites remained infectious for 9 months, and *E. coli* remained viable for 10 months. Protozoa genetic material and mRNA, as a sign of metabolic activity, were also assessed over time. The type of plant tilled under did not make a meaningful difference. Tilling contaminated crops into soil does not quickly eliminate pathogens. Some pathogens can survive for many months, meaning risks remain in fields for longer than expected.

## INTRODUCTION

Soil plays a critical role in food systems, acting not only as the foundation of plant growth but also as a potential reservoir for foodborne pathogens (1). Protozoan parasites, such as *Cryptosporidium parvum* and *Cyclospora cayetanensis*, are among the most concerning soil-associated pathogens, being highly persistent in agricultural environments (2) and capable of causing widespread outbreaks of foodborne illness (3). These parasites possess robust oocysts, which offer the organisms protection from environmental factors (4) as well as resistance to commercial sanitizers (5), complicating efforts to control their introduction into food systems. Similarly, shigatoxigenic varieties of *Escherichia coli* (STEC) are bacteria of significant public health concern; they are well studied and known to survive in soil for long periods of time (6) and are frequently implicated in outbreaks linked to contaminated produce (7). The overlapping challenges these organisms present highlight the need for a deeper understanding of their behavior in soil in order to develop more effective intervention strategies in the event of widespread contamination.

*Cryptosporidium* species, *C. parvum* and *C. hominis*, pose a relevant public health concern, related to waterborne infections and contaminated raw and ready-to-eat foods. From 2009 to 2017, *C. parvum* was implicated in 444 outbreaks in the United States, leading to 7,465 reported illnesses (8). *Cryptosporidium* is a protozoan pathogen of global importance, yet there remain ongoing food safety challenges associated with epidemiological and detection limitations (9), particularly in the fresh produce sector. *C. cayetanensis* has become an emerging threat, with a marked increase in outbreaks linked to fresh produce between 2018 and 2023 (3). Notable examples include a 2020 outbreak involving bagged salad mixes that caused 1,241 illnesses and a 2022 outbreak tied to leafy greens with 1,129 cases (3). The economic burden of such events is significant, including expenses related to recalls, lost revenue, and healthcare costs (10). Research on *C. cayetanensis* is hindered by its human host specificity, the difficulty of obtaining viable oocysts, and the lack of a functional cell culture model (11). Research on *C. cayetanensis* is hindered by the difficulty of obtaining oocysts from infected humans and the lack of a model effective to propagate the parasite outside the human host (12). The use of surrogates, such as *Eimeria tenella*, which is zoonotic and shares certain biological and genetic features with *C. cayetanensis*, has been instrumental in assessing the efficacy of sanitizers (11, 13).

Pathogenic strains of *E. coli*, such as *E. coli* O157:H7, continue to be some of the most significant foodborne pathogens globally, frequently linked to outbreaks associated with fresh produce and other agricultural products (14). Recent outbreaks, such as the outbreak in 2024, where 104 people were infected after consuming onions served at McDonald’s (15), emphasize the ongoing public health threat posed by this bacterium. *E. coli* spp. are well documented in their ability to persist in soil and other agricultural environments, surviving for several months under diverse environmental conditions such as temperature, moisture, and nutrient availability (16). Like protozoan parasites, soil-mediated contamination of produce with these pathogens can occur directly, through plant contact with contaminated soil (17), or indirectly, when irrigation water becomes contaminated via runoff, as in the case of adjacent land use (18). *E. coli* is one of the most extensively studied and well-understood foodborne pathogens (19), and assessing its persistence alongside the more challenging protozoan parasites will allow for existing knowledge to be leveraged in order to better understand microbial behavior in agricultural contexts.

Growers employ various strategies to mitigate the contamination of crops with foodborne pathogens, including the use of chemical sanitizers, the removal of debris via washing, and agricultural practices such as precision irrigation and the use of buffer zones, which reduce pathogen transfer from the surrounding environments (20). However, these methods each face unique limitations and are of little use when large swaths of crops are contaminated pre-harvest, as in the case of widespread animal intrusion. In such events, growers traditionally attempt to remediate the contamination by leaving affected crops unharvested and “tilling them under” (21, 22). Conventional wisdom dictates that the tilling process (i.e., burying the contaminated crops underneath a layer of soil and allowing them to decay over time) will cause the pathogens to die off; however, little research has been done to determine the rates at which certain pathogens, such as protozoan parasites, decay, or the dynamics of organism decay in relation to environmental conditions and the chemical characteristics of the soil itself.

Current methods for detecting and quantifying the number of viable, infectious oocysts of protozoan parasites in agricultural matrices are labor-intensive and complex (23). Following intricate multi-step recovery and isolation steps, the oocysts must be placed into mammalian cell cultures to trigger excystation and allow for the viable sporozoites to invade. After infection, the cells are recovered, qPCR is performed, and calculations are done to estimate the number of viable oocysts present in the original sample (24). While effective, these processes require special laboratory facilities, personnel with unique specializations, and considerable time. Additionally, the parasite *C. cayetanensis* only infects humans and currently has no known cell culture method (11), thus making reliable detection even more challenging. As an alternative, detecting the mRNA transcription products of well-characterized and highly conserved genes hsp70 (heat shock protein 70) in *C. parvum* (25) and hsp90 in *E. tenella* (26) may offer a promising solution. Unlike DNA, which persists even in dead oocysts, mRNA is only produced by metabolically active organisms, giving it the potential to act as a marker for live protozoa (27). This approach sidesteps the need for mammalian cell culture, hastening and simplifying the workflow while maintaining accuracy and potentially providing a viable method for the detection of organisms like *C. cayetanensis*.

This study aims to address critical knowledge gaps that exist regarding how foodborne pathogens, including *C. parvum*, *C. cayetanensis* (using *E. tenella* as a surrogate), and *E. coli,* behave on plant tissue as it decays following a simulated “tilling under” process. Specifically, this study will monitor the infectivity of these organisms as they persist on decaying plant tissues, assess whether different types of leafy green tissue result in different organism die-off dynamics, and evaluate the influences of soil chemistry and environmental factors on organism persistence. This study will also determine if monitoring the decay of mRNA transcripts can serve as a reliable alternative to traditional infectivity assay methods, offering a culture-independent method to evaluate the risks associated with protozoan parasites. Through these objectives, this study will provide actionable insights into the efficacy of the traditional soil-management practice of tilling under, thus allowing for a deeper understanding and improved application of this method.

## MATERIALS AND METHODS

### Organism acquisition

Live *C. parvum* oocysts (Iowa isolate) were sourced from Waterborne Inc. (New Orleans, LA, USA), where they were propagated in experimentally infected calves. The oocysts were suspended in PBS containing 0.01% Tween 20 and supplemented with antibiotics and antifungal agents, including penicillin, streptomycin, gentamicin, and amphotericin B, to maintain viability and sterility. Prior to use, the oocysts were refrigerated at 4°C and utilized within 2 months of receipt. *E. tenella* oocysts were generously supplied by the USDA-ARS Animal Parasitic Diseases Laboratory (Beltsville, MD, USA), where they were propagated in experimentally infected poultry. The oocysts were collected from the ceca, thoroughly washed, and preserved in 2% potassium dichromate at 4°C. Experimentation began within 1 month of receiving the oocysts. At the time of initial sample preparation, the oocysts of both *C. parvum* and *E. tenella* were at least 90% sporulated, as determined by microscopy. *E. tenella* was employed as a surrogate for *C. cayetanensis* in this study, due to biological similarities such as analogous biosynthesis pathways and production of double-layered oocysts and sporocysts that enclose pairs of sporozoites, as well as the oocyst’s intricate surface proteins critical to excystation and cellular invasion (11, 28).

*E. coli* TVS355 is a non-pathogenic strain originally isolated from environmental soil (29); it was selected for this study due to its frequent use as a surrogate for *E. coli* O157:H7 in field and greenhouse experiments (30). The inoculum was prepared following a modified version of the protocol described (31). To differentiate the inoculum from background flora, *E. coli* TVS355 was adapted to grow in the presence of 80 µg/mL rifampicin, as previously described (32). The strain was revived from stock stored at −80°C by streaking onto tryptic soy agar (TSA) plates, followed by incubation at 37°C for 24 h. A single colony isolate was then transferred into a 50 mL conical tube containing 40 mL of tryptic soy broth (TSB), which was then incubated at 37°C for another 24 h. The resulting broth culture was enumerated by serial dilution and plating onto TSA, and a final inoculum of 9 log CFU/mL was prepared by diluting the appropriate volume of broth culture into 100 mL of 0.1% buffered peptone water (BPW). The samples were inoculated with 100 μL of this culture, resulting in 10^8^ CFU of bacterial culture being added to each sample.

### Soil sample preparation

Sandy loam topsoil (5 in.–6 in.) was collected from the US Department of Agriculture, Agricultural Research Service, Beltsville Agricultural Research Center (USDA-ARS-BARC) North Farm (33). Samples were sent to the University of Delaware Soil Testing Laboratory for Mehlich 3 extractable nutrient analysis before use in experimentation; the soil was then distributed in 120 g aliquots into 4 oz standup sampling bags (*n* = 156 bags). Three types of samples were prepared for this experiment (*n* = 52 per sample type): soil containing basil; soil containing cilantro; and soil containing no tissue (referred to as NT soil). Fresh basil and cilantro were procured from a local distributor; 6 g portions of plant tissue, containing both stem and leaf, were then weighed into individual sterile petri dishes. Each sample was inoculated dropwise with 10^6^ oocysts of *C. parvum*, 10^6^ oocysts of *E. tenella*, and 10^8^ CFU of *E. coli* TVS355, and then allowed to air dry for 120 min to allow for organism adhesion. One plant sample was then added to each soil bag, and the samples were massaged by hand until the plant tissue was at a depth of 17 cm (approximately 2 min). This depth is considered “shallow inversion tillage,” a method which has demonstrated comparable results in terms of overall produce yield relative to deep tillage while also resulting in higher soil carbon and enhanced weed control as compared to other tillage methods (34). NT soil samples were prepared by inoculating each organism directly into 120 g of soil; the sample was then set aside for 120 min and then massaged by hand for 2 min in the same manner as the samples containing plant material. Half of the samples were then placed in a 12°C incubator, and the other half were placed in a 32°C incubator. These temperatures were selected as being representative of the expected lows and highs observed throughout the Delaware growing season, also representative of the Mid-Atlantic area of the United States (35). After 4 and 11 months of incubation, samples (*n* = 4) of each type were again sent for soil testing to monitor changes in extractable nutrient compositions over time. Throughout the experiment, soil moisture content was measured using a Digital Soil Moisture Meter (DSMM500) with an 8 in. probe (General Tools, Secaucus, NJ, USA).

### Recovery of organisms

On a monthly basis, six samples (two replicates per sample type, including the no tissue control) were removed from each incubator and evaluated for organism survival. Thirty grams of each sample was dispensed in duplicate into 24 oz filtered whirlpak sampling bags; 120 mL of 1× PBS supplemented with 30 g/L of beef extract was then added to each, and the bags were homogenized by hand for 2 min. For bacterial enumeration, 5 mL aliquots were taken from the duplicate bags and combined in 15 mL conical centrifuge tubes. These aliquots were then serially diluted in BPW and plated onto MacConkey agar supplemented with 80 µg/mL rifampicin.

Protozoan parasites were recovered from the remaining 230 mL of media contained in the duplicate sampling bags. The method was adapted from the US Food and Drug Administration Bacteriological Analytical Manual, chapter 19C (36). Briefly, the contents of the duplicate bags were evenly distributed into eight 50 mL conical centrifuge tubes and centrifuged at 2,000 × *g* for 20 min. The supernatants were carefully decanted, and the pellets were resuspended in 2% bleach solution to reduce background microbial populations. The contents of all eight tubes were then combined into two 50 mL conical tubes and centrifuged as before. The supernatants were removed, and the pellets were each resuspended in 5 mL of sterile 1× PBS and combined; the contents of the eight original tubes now being in a single 50 mL tube. This tube was centrifuged at 2,000 × *g* for 20 min, the supernatant was removed, and the pellet was resuspended in 10 mL of PBS before undergoing another round of centrifugation. The tube was then decanted and underwent one final round of resuspension in 10 mL of PBS and centrifuged at 12,000 × *g* for 3 min. The resulting pellet was suspended in 2 mL of 1× PBS for use in infectivity assays and mRNA quantification.

### Detection—infectivity paired with qPCR

The infectivity of *C. parvum* oocysts was assessed using human ileocecal adenocarcinoma cells (HCT-8 cells, ATCC CC-244, American Type Culture Collection, Manassas, VA, USA) following previously described methodologies (37). Prior to use in experimentation, cells were cultured in RPMI-1640 medium (Mediatech Inc., Manassas, VA) supplemented with 10% fetal bovine serum (FBS) for growth or 2% FBS for maintenance, along with 100 U/mL penicillin G, streptomycin, and amphotericin B, and maintained in a humidified incubator at 37°C with 5% CO_2_. *E. tenella* oocysts were assessed in their ability to infect Madin-Darby bovine kidney cells (MDBK cells, ATCC NBL-1, American Type Culture Collection, Manassas, VA), cultured in DMEM (Mediatech Inc., Manassas, VA) supplemented with 10% FBS for propagation or 2% FBS for maintenance, and grown under 37°C and 5% CO_2_; methods were derived from reference 38.

Following recovery and washing, 500 µL of the resuspended pellet was applied to 85%–95% confluent HCT-8 or MDBK cells grown in six-well plates. The plates were incubated at 37°C for 60 min to facilitate oocyst excystation. The inoculum was removed, 2 mL of maintenance media was added, and the plates were returned to incubation, allowing time for any remaining viable sporozoites to infect the cells. After 48 h, the infected cells were recovered by removing the maintenance media and replacing it with 0.25% trypsin (500 µL per well); the plates were then incubated for 10 min to allow the cells to detach. The infected and now detached cells were collected and stored for later nucleic acid extraction. In each experiment, infectivity of untreated inoculum was assessed alongside uninoculated in separate wells.

Nucleic acid extractions were conducted on the collected cells using the Zymo Quick-DNA MiniPrep kit (Zymo Research, Irvine, CA, USA), following the “Cell Monolayer Sample” protocol outlined in the user manual. Triplicate qPCR assays were performed for each sample, including positive controls and no-template controls. Primer sequences targeting the *C. parvum* CPHSPT2F gene (346 bp target) were previously reported (37), while primers specific to the *E. tenella* 5S rRNA gene (278 bp target) were described elsewhere (26, 39). PCR analysis was performed as described in Craighead et al. (37), with a total reaction volume of 25 µL, containing 12.5 μL of Rotor-Gene SYBR Green PCR master mix or QuantiNova Probe PCR master mix (Qiagen, Germantown, MD), 1 μL (400 nM) of each primer, forward and reverse, 1 μL (200 nM) of probe, 8 μL of DNA template, and 1.5 μL–2.5 μL of nuclease-free water. All qPCRs were conducted using a Rotor-Gene Q Thermocycler (Qiagen, Hilden, Germany) using the following cycling parameters: initial hold/heat inactivation at 95°C for 5 min, 40 cycles of denaturation and combined annealing/extension at 95°C for 5 s and 60°C for 10 s, with signal acquisition during combined annealing/extension. Details on previously described primer sequences are provided in Table 1.

**TABLE 1:** Primers and probes used for the detection of parasite nucleic acid

Experimental target	Oligo	Primer/probe sequences (5′–3′)	Genomic target
*C. parvum* DNA post-infectivity	Forward	TCC TCT GCC GTA CAG GAT CTC TTA	CPHSPT2F (346 bp target)
	Reverse	TGC TCT TAC CAG TAC TCT TAT CA	
*E. tenella* DNA post-infectivity	Forward	TCA TCA CCC AAA GGG ATT	5s rRNA (278 bp target)
	Reverse	TTC ATA CTG CGT CTA ATG CAC	
	Probe	Fam-CGC CGC TTA ACT TCG GAG TTC AGA TGG GAT-Tamra	
*C. parvum* mRNA	Forward	CGTA ATA CAA CTA TCC CAG CAA	Hsp70 (189 bp target)
	Reverse	CAA TAT CAA AGG TGA CTT CAA TTT	
	Probe	Fam-CAG AGT GGT-/Zen/-GTC TTG ATC CAA GTT T	
*E. tenella* mRNA	Forward	GTG AAG GGT GTT GTT GAC TC	Hsp90 (~200 bp target)
	Reverse	AGT CTG ACG AGA CTC TCC AG	

For use in qPCR as a standard curve, HCT-8 and MDBK cells were also infected with 10^6^ fresh, sporulated oocysts following the protocol described above. This serves as the positive control, and the negative uninoculated control was processed alongside this. The recovered monolayers underwent nucleic acid extraction, which was then serially diluted 10-fold and analyzed alongside genetic material from oocysts that underwent the experimental protocol. The infectivity assay followed by qPCR quantifies the number of oocysts that remain infectious on decaying plant tissue in soil for extended periods of time. Non-infectious or non-excysting oocysts are removed during washing, ensuring that only oocysts capable of invading the cell culture are collected after trypsinization. The limit of detection for this assay was 16 genomic copies via qPCR (40), with a Ct cutoff of 40 cycles.

### Detection—mRNA decay method

After recovering the oocysts from soil samples, the resulting suspensions were immediately frozen at −80°C, preventing further expression of gene products and preserving any mRNA already present (41, 42). The oocysts were thawed and had their total RNA extracted, and the resulting mRNA was then immediately converted to cDNA to be quantified via RT-qPCR. Briefly, a 500 µL portion of the thawed oocyst suspension was added to Qiagen PowerBead Tubes containing ceramic 1.4 mm beads (Qiagen Inc., Hilden, Germany); 500 µL of Trizol (Invitrogen, Carlsbad, CA, USA) was also added, and the tube was vortexed at 4,000 rpm for 5 min. Tubes were centrifuged at 10,000 × *g*, and the supernatant was transferred to a new nuclease-free tube. The remaining steps of the RNA extraction were carried out using the Zymo Direct-Zol RNA MiniPrep kit (Zymo Research, Irvine, CA, USA) following the manufacturer’s instructions. Extracted mRNA was converted into cDNA using the QuantiTect Reverse Transcription Kit (Qiagen Inc., Hilden, Germany) following the provided instructions. The cDNA was amplified through qPCR, allowing quantification of the *C. parvum hsp*70 and *E. tenella hsp*90 genes. Table 1 provides the specific primer sequences used in this study (26, 37, 39). Cycling parameters for *C. parvum hsp*70 cDNA amplification were as follows: hold at 95°C for 10 min, 40 cycles of denaturation and combined annealing/extension at 95°C for 5 s, 55°C for 15 s, and 72°C for 8 s; final extension was performed at 72°C for 10 min, and signal was acquired during annealing/extension. *E. tenella hsp*90 cDNA amplification was performed using the following cycling parameters: hold/heat inactivation at 95°C for 7 min, denaturation at 94°C for 30 s, annealing at 65°C for 30 s, extension at 72°C for 60 s, and a final extension at 72°C for 5 min. Denaturation, annealing, and extension steps were repeated 40 times, and signal was acquired during the extension step.

In addition to the recovered oocysts, mRNA was extracted from 10^6^ sporulated oocysts of *C. parvum* and *E. tenella*. This mRNA underwent the same cDNA synthesis protocol previously mentioned; it was then serially diluted 1:10 and evaluated in RT-qPCR alongside the samples in order to generate a standard curve. The mRNA products of these genes decay rapidly and should only be present when there are live organisms present and expressing target genes (27). Therefore, detecting these gene products in a sample should indicate the presence of live organisms, and measuring the abundance of these products in comparison to a standard curve should allow for the quantification of these organisms.

### Data analysis

Throughout the study, experiments were conducted in duplicate, with molecular detection and bacterial enumeration being performed in triplicate (*n* = 6 per sample). Data analysis and figure generation were done using JMP Pro 16 and Microsoft Excel statistical software. One-way analysis of variance, Tukey’s HSD, and Student’s *t*-test were used to calculate significance and correlations between variables and organism survival. *P*-values less than 0.05 were considered statistically significant, with an alpha level of 0.05 (α = 0.05) and a confidence interval of 95%.

## RESULTS

### Soil analysis results

After 4 and 11 months of incubation, the quantities of soil nutrients did not change significantly (*P* > 0.05), and the quantities of nutrients were not significantly (*P* > 0.05) different between sample types (basil, cilantro, or NT soil) or incubation temperatures (12°C and 32°C). The nutrients measured, as well as the concentrations (average amounts measured across all samples evaluated, with standard deviations in parentheses) are phosphorus 382.9 (±12.58) mg/kg, potassium 114.6 (±4.39) mg/kg, calcium 891.6 (±37.01) mg/kg, magnesium 98.5 (±5.27) mg/kg, manganese 15.1 (±1.79) mg/kg, zinc 5.7 (±0.31) mg/kg, copper 1.1 (±0.07) mg/kg, iron 575.8 (±9.3) mg/kg, boron 0.1 (±0.04) mg/kg, sulfur 33.1 (±2.61) mg/kg, sodium 33.1 (±8.72) mg/kg, and aluminum 613.9 (±26.81) mg/kg. Soil moisture content decreased gradually over time. When incubation began, the samples had a moisture content of approximately 26%. After a period of 4 months, this decreased to 23%. After 12 months, the samples incubated at 12°C had a moisture content of 21%, which was significantly (*P* < 0.05) different from the 18% moisture content of samples incubated at 32°C.

### *E. coli* TVS 355 decay

**Fig 1:**
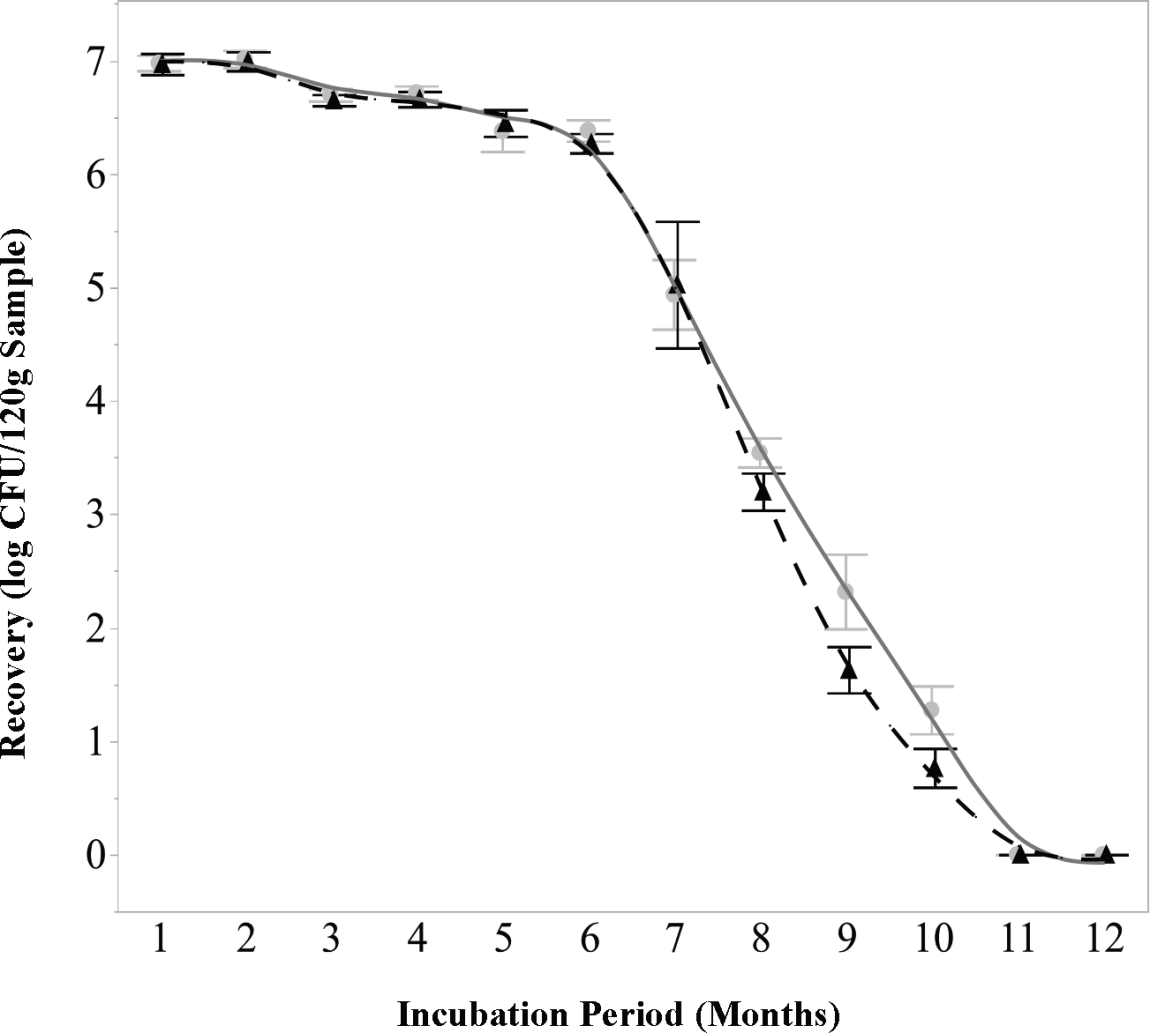
*E. coli* recovery over time by incubation temperature. *E. coli* remained detectable for 10 months, exhibiting slow initial decline followed by rapid die-off after 6 months. After 8 months, significantly (*P <* 0.05) higher recovery was observed in samples incubated at 12°C as compared to 32°C. Sample type (basil, cilantro, or NT soil) had no significant (*P >* 0.05) effect on recovery; data represent the mean *E. coli* recovered across all three sample types (*n* = 6). Circle and solid line represent samples incubated at 12°C; triangle and dashed line represent samples incubated at 32°C.

The amount of culturable *E. coli* in the samples decayed gradually over time, with insignificant (*P* > 0.05) differences in recovery between basil, cilantro, and NT soil samples, and significant (*P* < 0.05) differences in recovery between the two temperatures, 12°C and 32°C, appearing only after 8 months of incubation (Fig. 1). *E. coli* died off slowly initially, with an average of 6.35 log CFU/sample being recovered from the 8 log CFU/sample starting inoculum after 6 months of incubation. There was a dramatic reduction in recovery after 7 months, with 4.48 log CFU/sample being recovered at this time, with subsequent month-to-month reductions, each being nearly as large. After 8 months of incubation, a significantly (*P* < 0.05) greater amount of *E. coli* was detected in samples incubated at 12°C (3.55 log CFU/sample) as compared to those at 32°C (3.2 log CFU/sample). The significant (*P* < 0.05) differences in recovery between temperatures were greatest after 9 months, where 2.32 log CFU/sample was measured at 12°C, and 1.63 log CFU/sample was measured at 32°C. *E. coli* was last detected after 10 months, with 1.28 log CFU/sample recovered at 12°C and 0.76 log CFU/sample recovered at 32°C.

### Protozoa decay

As described below, there is no significant difference (*P* > 0.05) in the number of protozoa across these types of matrices. For example, after 3 months of incubation, on average, 5.12 log oocysts were recovered from samples with cilantro or basil tissue, and on average, 5.16 log oocysts were recovered from samples without plant tissue.

#### *C. parvum* decay

**Fig 2:**
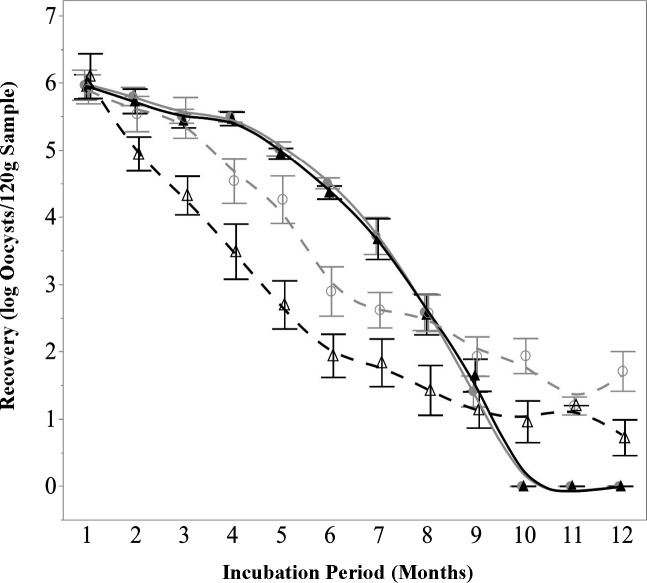
*C. parvum* detection over time with respect to temperature and method. *C. parvum* oocysts exhibited a gradual decline in infectivity, with the last detectable infection of HCT-8 cells occurring at 9 months and hsp70 mRNA remaining detectable through 12 months. Sample type did not significantly (*P* > 0.05) affect detection for either method; data represent the mean number of oocysts detected across all three sample types (basil, cilantro, and NT soil) (*n* = 6). Filled circle and solid line represent infectivity, incubated at 12°C; filled triangle and solid line represent infectivity, incubated at 32°C; open circle and dashed line represent mRNA, incubated at 12°C; open triangle and dashed line represent mRNA, incubated at 32°C.

When measured via infectivity assay, the number of infectious *C. parvum* oocysts in the samples gradually declined over time (Fig. 2, filled symbols and solid lines). Insignificant (*P* > 0.05) differences in oocyst recovery were observed between sample types, as well as those incubated at 12°C and 32°C. As such, data described here represent combined averages of the recoveries at both temperatures and across basil, cilantro, and NT soil samples. Significant (*P* < 0.05) reductions in infectivity were first observed after 3 months, where 5.48 log oocyst/sample were detected out of the 6-log starting inoculum. After 9 months of incubation, an average of 1.53 log oocysts/sample was detected, with oocysts no longer being detectable by month 10.

The persistence of *C. parvum*, as measured by mRNA detection, showed a progressive decline over time (Fig. 2, open symbols and dashed line), with significantly (*P* < 0.05) fewer oocysts consistently being detected in samples incubated at 32°C, but insignificant (*P* > 0.05) differences being observed between sample types. Through the mRNA detection method, significant (*P* < 0.05) reduction was first observed after 2 months, with concentrations decreasing from the initial 6 log oocysts/sample to 5.54 log oocysts/sample detected in those incubated at 12°C and 4.95 log oocysts/sample at 32°C. The greatest difference in recovery between temperatures occurred after 5 months, where significantly (*P* < 0.05) more oocysts were found at 12°C (4.26 log) as opposed to 32°C (2.69 log oocysts/sample). By month 9, when the oocysts were no longer detectable via mammalian cell culture methods, recoveries had declined to 1.93 log oocysts/sample at 12°C and 1.14 log oocysts/sample at 32°C. Oocysts remained detectable throughout the experiment at both temperatures; after 12 months of incubation, 1.71 log oocyst/sample were detected in those incubated at 12°C, and 0.72 log oocysts/sample were detected in those incubated at 32°C.

#### *E. tenella* decay

**Fig 3:**
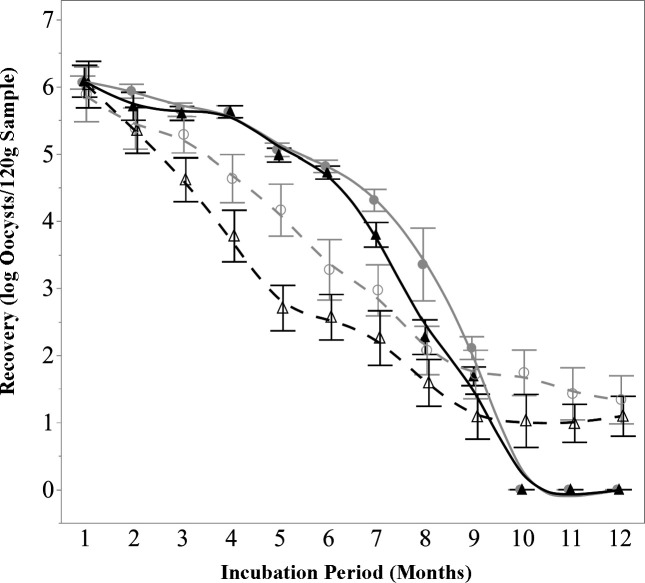
*E. tenella* detection over time with respect to temperature and method. *E. tenella* oocysts progressively lost infectivity over time, with the last detectable infection of MDBK cells occurring at 9 months, though hsp90 mRNA remained detectable for at least 12 months. Sample type had no significant effect on detection for either method; data represent mean numbers of oocysts across all three sample types (basil, cilantro, and NT soil) (*n* = 6). Filled circle and solid line represent infectivity, incubated at 12°C; filled triangle and solid line represent infectivity, incubated at 32°C; open circle and dashed line represent mRNA, incubated at 12°C; open triangle and dashed line represent mRNA, incubated at 32°C.

When monitored through traditional infectivity-based methods, the rate of *E. tenella* decay started out slowly, but increased over time (Fig. 3, filled symbols and solid lines). There were no significant (*P* > 0.05) differences in recovery between sample types; however, there were significant (*P* < 0.050 differences attributable to incubation temperature after the first several months. As compared to the 6 log oocyst/sample starting inoculum, significant (*P* < 0.05) reductions were first observed after 3 months of incubation, at which point an average of 5.63 log infectious oocysts/sample were detected. After 7 months, significantly (*P* < 0.05) fewer infectious oocysts were detected in samples incubated at 32°C as compared to 12°C, 3.36 log and 3.8 log oocysts/sample, respectively. Nine months was the final time point at which oocysts were detectable through infectivity. In samples incubated at 12°C, 2.11 log oocysts/sample were detected, and in samples incubated at 32°C, 1.69 log oocysts/sample were detected.

The number of *E. tenella* oocysts measured in the samples, as determined by mRNA detection, declined over time (Fig. 3, open symbols and dashed line). The differences in *E. tenella* detection were insignificant (*P* > 0.05) between the different sample types; however, there were significant (*P* < 0.05) differences in *E. tenella* mRNA concentration between the two incubation temperatures after 3 months and through the end of the experiment. After 2 months of incubation, the number of oocysts decreased significantly (*P* < 0.05), from the starting 6 log oocysts/sample to 5.36 log oocysts/sample. After 3 months, significantly (*P* < 0.05) greater numbers of oocysts were detected in 12°C samples (5.29 log oocysts/sample) as compared to 32°C samples (4.62 log oocysts/sample). The greatest differences in recovery between the two temperatures were seen after 5 months of incubation, when 4.17 log oocysts/sample were detected at 12°C, and 2.71 log oocysts were detected in 32°C samples. At month 9, when *E. tenella* was last able to infect mammalian cell culture, mRNA methods detected 1.72 log oocysts in 12°C samples, and 1.09 log oocysts in 32°C samples. *E. tenella* continued to be detectable through the end of the experiment, with 1.34 and 1.09 log oocysts/sample being measured at 12°C and 32°C after 12 months of incubation.

## DISCUSSION

Previous research has demonstrated that different types of *E. coli* are able to survive for long periods of time under a variety of environmental circumstances both in-field and under laboratory conditions (16). In this study, *E. coli* TVS355 was able to persist on decaying plant tissue in soil, following a simulated tilling-under process, for a period of 10 months. These results are most similar to those previously reported in reference 30, where a cocktail of *E. coli* strains (including TVS355) was able to remain cultivable for a period of 336 days in amended soil samples with moisture contents ranging from 12.5% to 22.2%. These findings reinforce the extensive body of research demonstrating the resilience of *E. coli* in soil environments and validate its use as a well-characterized model organism for microbial persistence studies. However, the differences in the die-off rates of protozoa between detection methods (infectivity vs mRNA) suggest that attempting to use *E. coli* as an indicator for protozoal decay may only offer insight into the rate at which protozoa lose their ability to infect mammalian cell cultures rather than how long it takes for these organisms to no longer be detectable, or potentially pose a contamination risk, in agricultural soil following widespread contamination.

Protozoan persistence in this study is consistent with those from Rogers et al. (43), also evaluating protozoan decay in soil and on fresh herbs, where oocysts of *C. cayetanensis* remained detectable on herb leaves for at least 56 days in some instances, with moisture appearing to serve as the main contributor toward persistence (as opposed to temperature or soil type). While the previous study detected consistent quantities of oocysts throughout the experiment on plant tissue, it was noted that oocysts in soil that did not receive regular watering became undetectable after 21 days, while those in wet conditions remained detectable longer. These observations align with the findings of the present study, which are that environmental conditions can significantly influence the persistence of protozoan oocysts and detection of their genetic material, as evidenced by temperature-dependent mRNA degradation. Despite the differences in organisms studied and detection methods, both studies highlight the importance of environmental conditions in determining oocyst decay, with moisture potentially being a driving factor in persistence and detection of protozoan oocysts.

Although *C. parvum* is generally believed to be more susceptible to environmental stressors than *E. tenella* (13), their similar survival in this study (as determined by infectivity-based methods) suggests that both can persist equally well in undisturbed soil. The sporocysts and thicker-walled oocysts of *E. tenella* (characteristics which are shared by *C. cayetanensis*) may provide greater protection, while *C. parvum* oocysts, with thinner walls and lack of sporocysts, are more vulnerable to external stressors (44); however, without sanitation treatments or other disturbances, *C. parvum* oocysts may enter a relatively stable state, allowing them to persist similarly to oocysts of *E. tenella*. Furthermore, *E. tenella* showed greater temperature sensitivity, with higher recovery at 12°C than 32°C, while *C. parvum* exhibited consistent decay at both temperatures. This may be due to the ability of *E. tenella* to sense environmental conditions via trans-surface structures (45, 46), allowing the organism to delay excystation until favorable conditions arise. In contrast, *C. parvum* relies on a simpler suture-like structure to facilitate excystation (44), leading to a more uniform decay pattern across temperatures over time. These findings highlight the role of oocyst conformation in persistence and the need for well thought-out intervention strategies in food safety that are specifically tailored to the organisms of concern. Recall that in this study, information is sought that can be applied toward our understanding of *Cyclospora* oocysts. Despite the similarities of *Eimeria* and *Cyclospora*, which are noted throughout this study, differences between the organisms exist which may influence their responses to environmental conditions. Notably, sporulation in *Eimeria* species occurs rapidly, within 24 to 48 h, compared to the 7 to 15 day sporulation period typical of *C. cayetanensis*. Sporulated *Eimeria* oocysts also contain four sporocysts, whereas *C. cayetanensis* oocysts contain only two (28). These distinctions could potentially impact the translatability of results obtained with *E. tenella* as a surrogate for *C. cayetanensis*.

When using traditional infectivity-based methods to monitor protozoal decay, *C. parvum* and *E. tenella* both remained infectious in soil for a period of 9 months, while *E. coli* TVS355 outlived both parasites, remaining culturable for 10 months. In contrast, when using mRNA methods, oocysts of *C. parvum* and *E. tenella* outlasted *E. coli* TVS355, remaining detectable at low levels for at least 12 months. The present study also found that, through infectivity methods, differences in soil composition, ambient temperature, and the type of plant tissue that was tilled generally do not appear to impact the die-off of these organisms (albeit, with *E. tenella* showing greater sensitivity to high temperatures than *C. parvum* and *E. coli* after several months of incubation). Meanwhile, mRNA methods indicated that temperature impacts oocyst persistence almost immediately, with far fewer oocysts of both species being detected in samples incubated at the higher temperature. This heightened temperature sensitivity may be attributed to the inherent instability of mRNA molecules, which are highly susceptible to degradation under elevated temperatures due to increases in enzymatic activity (47), unlike DNA molecules, which are much more durable and can be detected even in fully inactivated oocysts (48, 49). The benefit of this observed sensitivity is that the detection of these mRNA molecules should indicate the presence of live organisms that are undergoing even low levels of enzymatic activity.

Unlike infectivity assays, which assess the ability of an organism to successfully invade mammalian cell cultures, mRNA quantification reflects the presence of genes that are being actively transcribed; however, throughout an organism’s life, certain genes may be differentially expressed (50). The life cycles of protozoan parasites are complex (and in the case of *C. cayetanensis,* not well understood), with the organisms moving through several distinct physiological states with unique gene expression profiles (25) and even possessing the ability to enter dormancy while maintaining the ability to cause infection (51). These differences in gene expression are a confounding factor when attempting to quantify oocyst abundance through mRNA methods and highlight the need for candidate genes to be highly conserved and resistant to unpredictable fluctuations in expression. Identifying such genes is critical for developing reliable molecular methods to assess the persistence of protozoan parasites in growing environments. Doing so will be particularly useful for organisms like *C. cayetanensis*, which is of growing concern in the United States and lacks infectivity-based methods (11).

The *hsp*70 and *hsp*90 genes in *C. parvum* and *E. tenella* used in this study are primarily responsible for maintaining homeostasis under thermal pressures, though they have also been shown to be upregulated under certain conditions—*hsp*70 in response to oxidative stress (52) and *hsp*90 just prior to cellular invasion (53). Outside of these upregulating conditions, these genes are understood to be expressed at relatively stable levels in the oocyst phase of the life cycle (52, 53); this stability makes them valuable targets for molecular detection methods. Similar *hsp*70 homologs have been identified in *C. cayetanensis* (54), suggesting that an *hsp*70-based assay could potentially be adapted for this species; however, limited functional characterization and incomplete genome annotation make it difficult to confirm the consistency of its expression across life stages and in the presence of environmental stressors. The identification of similarly reliable markers *in C. cayetanensis* remains challenging due to limitations in available reference genomes and oocysts of the organism itself. Existing genome assemblies for *C. cayetanensis* are incomplete, with a large proportion of genes remaining uncharacterized. For example, the NCBI *C. cayetanensis* reference genome assembly CcayRef3 (NCBI RefSeq: GCF_002999335.1) contains 5,793 distinct protein-coding genes, of which 3,477 are classified as “uncharacterized” (55). In contrast, the NCBI reference genome for *C. parvum* ASM16534v1 (NCBI RefSeq: GCF_000165345.1) contains 3,805 protein-coding genes, none of which are uncharacterized (55). The lack of a well-annotated genome for *C. cayetanensis* presents a major hurdle in identifying candidate genes for viability assessment.

Despite these challenges, recent studies have successfully detected *C. cayetanensis*, even in low abundance, in agricultural samples using a primer set designated “Mit1C” which targets a portion of mitochondrial DNA that encodes cytochrome c oxidase subunit 3 (cox3) (56). The cox3 gene is known to be highly conserved within *C. cayetanensis*, and the Mit1C primer set has been demonstrated to be very specific, showing no cross-reactivity with other apicomplexans (56). However, little is known about the expression profile of cox3 throughout the *C. cayetanensis* life cycle or what factors may regulate its transcription. Given its conserved nature and what is currently known about the gene, further investigation into cox3 expression may reveal its potential as a stable molecular marker for oocyst viability. Consistent expression of cox3 mRNA across developmental stages and under environmental stressors could provide a foundation for culture-independent viability assessments, improving the detection and risk assessment of *C. cayetanensis* in food and environmental samples.

The discrepancies between infectivity and mRNA-based detection methods highlight the inherent complexities of assessing protozoan oocyst persistence and viability. Infectivity assays indicated that *C. parvum* and *E. tenella* oocysts were no longer capable of infecting mammalian cell cultures after 9 months, yet low levels of mRNA remained detectable at 12 months; this raises several possibilities regarding the fate of these oocysts. One interpretation is that the oocysts truly lost infectivity over time, and that the residual mRNA represents lingering transcriptional products from non-viable oocysts. This may suggest that mRNA within oocysts is more stable than previously thought, potentially preserved within the protective structures of the oocyst wall even after the organism has lost the ability to infect a host. Previous studies have shown that protozoan RNA decays rapidly after organism death, with an average half-life of up to 65 min depending on the life stage of the organism (47). However, if oocyst structures provide protection, this could extend the detectability of mRNA beyond expected degradation rates.

Alternatively, it is possible that the number of infectious oocysts declined below the threshold necessary to establish infection in *in vitro* assays, but some viable oocysts remained capable of causing infection *in vivo*, where host-specific factors might enhance their ability to excyst and invade. Given that *C. cayetanensis* infections often arise from low-dose exposures (57); this possibility is particularly concerning for food safety, as it underscores the limitations of infectivity assays in detecting low, yet still clinically relevant, levels of contamination. This theory somewhat conflicts with previous research, which found that oocysts of *E. tenella* became nonviable (i.e., unable to cause infection and propagate *in vivo*) after a period of 9 months (58); however, the same study found that other species of *Eimeria*, namely *E. maxima* and *E. acervulina,* all had markedly different decay profiles, with the latter even able to cause infection in chickens for up to 27 months under certain conditions (58). The distinct decay profiles between closely related protozoa emphasize a limitation of studies attempting to use surrogate organisms to predict the behavior of *C. cayetanensis*. Further research is needed to clarify the relationship between mRNA stability, oocyst viability, and the infectious dose required to pose a public health risk, particularly in the context of *C. cayetanensis*.

The persistence of genetic material in the absence of viable oocysts also raises important concerns regarding the interpretation of molecular detection results in agricultural or food production settings. Current practices for detecting *C. cayetanensis* rely on DNA targets (56), which are known to be significantly more durable than mRNA (48). If genetic material from *C. cayetanensis* remains detectable long after oocyst inactivation, there is a risk that molecular surveillance programs could yield positive results that may not necessarily translate to risk of infections. However, given that *C. cayetanensis* is a human-specific parasite, the detection of its DNA in fresh produce is inherently indicative of human fecal contamination at some point along the production or handling chain. Even in the absence of viable oocysts, such findings could therefore be considered an indicator of sanitation failure, which may warrant corrective actions. This could lead to unnecessary disruptions in food supply chains, including voluntary recalls or heightened concerns over potential foodborne outbreaks even in the absence of infectious agents. Such scenarios could have significant economic and public health implications, as they may undermine consumer confidence, place undue burdens on producers, and divert resources away from addressing actual contamination events. Distinguishing between the detection of non-viable genetic material and the presence of infectious oocysts is therefore critical for improving food safety risk assessments and ensuring that molecular detection methods provide accurate, actionable data for outbreak prevention.

This study demonstrated that, in instances of gross contamination, tilling under plant material can facilitate the decay of *E. coli, C. parvum*, and *E. tenella* (a surrogate for *C. cayetanensis*), supporting its potential as an effective microbial risk remediation strategy for agricultural soils. However, while extended field fallowing after tilling appears effective, its practicality may be limited, particularly for small growers for whom prolonged land idling is cost-prohibitive and impractical. These challenges emphasize the need for alternative or complementary strategies that balance pathogen reductions with agricultural productivity. It underscores key knowledge gaps in protozoan parasite research, specifically in detection and the understanding of *C. cayetanensis*, for which culture-based methods do not exist. With the knowledge gaps in mind, the careful selection of surrogate organisms remains critical, as differences in oocyst structures and environmental responses can influence experimental outcomes and limit the correlatability of results between organisms. This study also highlights the potential value of culture-independent detection methods for non-cultivable pathogens, though discrepancies between mRNA-based and infectivity-based results indicate that further research is required. Overall, advancing our understanding of these organisms and how they behave in soil will enable the development and application of more effective soil management strategies, thus improving food safety and reducing the public health risks associated with foodborne pathogens on produce.
